# Rare oncological diagnosis presenting as ‘rheumatic fever’

**DOI:** 10.1186/1546-0096-12-S1-P231

**Published:** 2014-09-17

**Authors:** Sunil Sampath, Mark Caswell, Liza J McCann, Clare E Pain, Eileen M Baildam, Michael W Beresford, Gavin Cleary

**Affiliations:** 1 Paediatric Rheumatology, Alder Hey Children's NHS trust, Liverpool, UK; 2 Paediatric Haematology, Alder Hey Children's NHS trust, Liverpool, UK; 3 Department of Women’s and Children’s Health, Institute of Translational Medicine, University of Liverpool, Liverpool, UK

## Introduction

CML (Chronic myeloid leukemia) is a myeloproliferative disorder, with dysregulated proliferation and differentiation of granulocytes. CML is rare during childhood, incidence of 1 in 1,000,000 and accounts for less than 5% of childhood leukemia. There are 3 different stages: chronic phase, accelerated phase and blast phase.

## Objectives

Discuss a case of CML, which presented as possible rheumatic fever.

## Methods

Case report and review of literature.

## Results

### Case report

A 13-year-old Caucasian boy, while on a skiing holiday in Switzerland, developed fever, pharyngitis, erythematous skin lumps and arthralgia; investigations revealed significantly raised Anti-streptolysin-O titres. His blood count showed a transient neutrophilia and thrombocytosis. General practitioner diagnosed him with streptococcal infection and treated him with oral Penicillin; he made full recovery in few days and returned to the UK. However he continued to have episodes of fever, arthralgia associated with raised inflammatory markers for the next 5 weeks. These episodes seem to occur every 10 days, lasting 4 – 5 days. In between these episodes he would make full recovery. He was continuing oral Penicillin prophylaxis and Anti-streptolysin-O titres were improving. Acute rheumatic fever was one of the differentials, however towards the end of ‘febrile episode’, he became neutropenic- which is not a recognised feature of acute rheumatic fever. Detailed investigations including: screen for common and rare bacterial and viral pathogens, biochemical profile, whole body isotope bone scan and urinary catecholamines, were within normal limits. Abdominal ultrasound revealed mild splenomegaly. Due to the neutropenia a bone marrow examination was performed, microscopy showed normal cellularity and maturation of the haematopoietic cells. Routine cutogenetic anaylysis was performed which demonstrated the classical Philadelphia chromosome t(9;22) BCR-ABL, thus supporting diagnosis of Chronic Myeloid Leukemia (CML). At presentation he was in the chronic phase.

## Discussion

In 2 recent case series of childhood CML, mean age at presentation was 11.5 years and 16 years. Chronic phase of CML is the most common type, seen in 92% childhood CML patients at presentation. Our patient presented with intermittent/periodic febrile episodes and neutropenia; these features were not described among a large case series of 430 CML patients (adult and childhood). In a childhood CML series, 2/13 children had fever as a presenting feature, the authors have not elaborated if this was periodic.

## Conclusion

To the best of our knowledge, intermittent/ periodic febrile episodes have not been described before as presenting features of childhood CML.

Bone marrow examination is highlighted as an important examination to consider in children presenting with pyrexias of unknown origin especially if associated with haematological abnormalities.

## Disclosure of interest

None declared.

